# The Role of Glial Fibrillary Acidic Protein in the Neuropathology of Alzheimer’s Disease and Its Potential as a Blood Biomarker for Early Diagnosis and Progression

**DOI:** 10.1007/s12035-025-05219-3

**Published:** 2025-07-21

**Authors:** Ekanayaka M. S. Bandara, Prita R. Asih, Steve Pedrini, Eugene Hone, Warnakulasuriya Mary Ann Dipika Binosha Fernando, Ralph N. Martins

**Affiliations:** 1 Centre of Excellence for Alzheimer’s Disease Research and Care, School of Medical and Health Sciences, Edith Cowan University, Joondalup, WA Australia; 2 Alzheimer’s Research Australia, Ralph and Patricia Sarich Neuroscience Research Institute, Nedlands, WA 6009 Australia; 3 Department of Biomedical Sciences, Macquarie University, Sydney, NSW 2109 Australia

**Keywords:** Alzheimer’s disease, AD, Glial fibrillary acidic protein, GFAP, Blood biomarkers, GFAP isoforms

## Abstract

Alzheimer’s disease (AD) is a neurodegenerative disease characterised by neuropathological hallmarks, including extracellular amyloid plaques and neurofibrillary tangles. The disease is clinically defined by cognitive dysfunction, including learning, memory deficits, and behavioural changes. With the rising global prevalence of AD, early diagnosis is critical for implementing effective interventions before irreversible neuronal damage occurs. Biomarkers correlating amyloid deposition, tau pathology, neuroinflammation, and neurodegeneration are currently being investigated using cerebrospinal fluid analysis and positron emission tomography imaging. These methods are invasive or costly, limiting their widespread clinical utility. Blood-based biomarkers offer a promising alternative due to accessibility, cost-effectiveness, and feasibility for large-scale screening. Among blood-based biomarkers, plasma glial fibrillary acidic protein (GFAP) levels have gained interest in identifying individuals at risk of AD at preclinical stages. However, significant challenges remain, including methodological inconsistencies, analytical variability, and the need for standardisation across immunoassay platforms to ensure the clinical applicability of plasma GFAP measurement in AD diagnosis. Additionally, the specificity of GFAP for AD needs further evaluation, as increased plasma levels are also observed in other diseases. Similar issues are found with p-tau 217, the blood biomarker candidate for AD that has received the most attention. This review summarises the role of GFAP in the neuropathology of AD, provides evidence on plasma GFAP as an early blood biomarker for AD and identifies key knowledge gaps that need to be addressed. Future advancements in assay development and large-scale longitudinal studies are essential to validate its diagnostic and prognostic potential for community-based AD screening.

## Introduction

Alzheimer’s disease (AD) is a progressive, neurodegenerative disorder characterised by the build-up of amyloid deposits and neurofibrillary tangles in the brain and clinically by cognitive decline, memory loss, and changes in behaviour and personality. Being the most common form of dementia, AD poses significant challenges to individuals, families, and healthcare systems worldwide. About 55 million people worldwide have AD [1]. The pathogenesis of AD is a complex, multifactorial process involving the interplay of genetic, environmental, and lifestyle factors [2, 3]. Major pathological hallmarks of AD are the accumulation of amyloid plaques in the brain parenchyma and intra-cellular aggregation of hyperphosphorylated microtubule-associated tau proteins as neurofibrillary tangles (NFT) [4–11]. At early stages, amyloid β (Aβ) plaques develop in basal, temporal, and orbitofrontal neocortex regions of the brain and eventually progress to the neocortex, hippocampus, amygdala, diencephalon, and basal ganglia. Although it is unclear where tau deposition originated, it has been postulated that Aβ can trigger NFT formation in the locus coeruleus, transentorhinal, and entorhinal areas of the brain [8]. The interplay between these pathological hallmarks, along with synaptic dysfunction and the loss of neuronal connectivity, culminates in progressive cognitive decline and dementia. Recent research also highlights the roles of neuroinflammation, driven by activated microglia and astrocytes, and the potential contributions of vascular dysfunction and metabolic disturbances, adding further complexity to the pathogenesis of AD [8–12].

The recently introduced drugs Aducanumab and Lecanemab are designed to target the underlying pathology of AD by reducing amyloid plaques. However, the primary benefit observed in clinical trials was a modest slowing of disease progression rather than a significant restoration of cognitive abilities [13–16]. Therefore, early diagnosis is crucial for managing AD, as it enables timely intervention and can potentially slow disease progression. However, the current diagnostic methods, which primarily rely on clinical assessment and neuroimaging, often detect the disease at an advanced stage when substantial neuronal damage has already occurred. In contrast, biomarkers such as Aβ positron emission tomography (PET) imaging can identify Aβ pathology in cognitively normal individuals, providing an opportunity for early detection and potential intervention [17]. This highlights the urgent need for reliable biomarkers for early and accurate diagnosis.

Preclinical diagnosis of AD is necessary to maximise the effectiveness of AD interventions, as biochemical changes begin at least 10–20 years before the onset of clinical symptoms [18, 19]. Recently, the Alzheimer’s Association Workgroup established objective criteria for diagnosis and staging AD by incorporating recent advances in biomarkers [19]. These updates reflect advancements in biomarker research and improve the accuracy of AD diagnosis by more effectively distinguishing early pathological changes.

However, PET scans are costly, and cerebrospinal fluid (CSF) collection for biomarker analysis is invasive, requiring a lumbar puncture to obtain a sample [20]. As a result, neither of these methods is practical for community-wide screening. In contrast, blood biomarkers offer a viable option for assessing AD risk and monitoring disease progression. Blood collection is a standard procedure that is both inexpensive and minimally invasive. This cost-effective collection method could serve as an alternative for AD-related biomarker analysis, addressing the limitations of brain imaging biomarkers [21].

Several promising blood-based biomarkers of AD have been evaluated as potential diagnostic tests, including Aβ peptides (Aβ_42_ and Aβ_40_), phospho-tau proteins (p-Tau 181, p-Tau 217, and p-Tau 231), and glial fibrillary acidic protein (GFAP). Notably, blood GFAP has emerged as a potential early biomarker for AD [22].

GFAP is an intermediate filament III protein found in the cytoskeleton of astrocytes, which are essential support cells in the nervous system. It plays a critical role in maintaining astrocytes’ mechanical strength and integrity, thereby supporting neurons and helping maintain the blood–brain barrier [23]. Astrocytes are involved in various vital functions, including neurodevelopment, synaptogenesis, and synaptic transmission. They provide metabolic support to neurons, regulate blood flow, maintain extracellular ion balance, and respond to injury [23–25].

Elevated levels of blood GFAP, which indicate astrocyte activation, have been observed in the preclinical stages of AD. This makes GFAP a promising predictive biomarker for future cognitive decline and dementia associated with AD [26–29].

Studies have shown that elevated blood GFAP levels are associated with Aβ plaque levels in the brain, suggesting that blood GFAP may help identify individuals with ongoing Aβ pathology at an early stage [22, 30]. Additionally, increased blood GFAP levels have been linked to cognitive decline and the prognosis of AD [28, 31]. Given these associations, measuring blood GFAP could serve as a potential screening tool to identify individuals at risk of AD, allowing for earlier intervention and management strategies for AD prevention. As a prognostic marker, blood GFAP may also be useful in monitoring disease progression and the effectiveness of therapeutic interventions. When combined with other well-established AD biomarkers, blood GFAP may enhance the accuracy and comprehensiveness of AD diagnosis and prognosis [32, 33].

This review provides a comprehensive analysis of the current research on plasma GFAP as a blood biomarker for AD screening. It explores the biological role of GFAP in AD, summarises key findings from recent studies examining GFAP levels in AD patients, and discusses the clinical implications, limitations, and future directions of using plasma GFAP in early diagnosis and prognosis of AD.

## Diagnosis of AD

Traditionally, the diagnosis of AD has required a comprehensive evaluation of clinical assessment and neuropsychological testing. More recently, neuroimaging and biomarker analysis have been playing a prominent role. Definite diagnosis of AD has been *post-mortem* histopathological examination demonstrating the presence of amyloid plaques and hyperphosphorylated tau tangles in the brain [34–41]. However, advancements in biomarker research now allow for a highly reliable diagnosis during life, mainly through CSF biomarkers or PET imaging of amyloid and tau pathology. Blood biomarkers are largely still in development, though one company in the US is currently offering a blood test, and several companies will be offering blood tests in the near future.

To date, AD diagnosis during life is primarily based on established clinical and research criteria such as those established by the National Institute on Aging and the Alzheimer’s Association (NIA-AA) (2011), International Working Group (IWG) (2010) [42–45], and the *Diagnostic and Statistical Manual of Mental Disorders (DSM) Fifth Edition* criteria [46]. These criteria emphasise the need for a detailed medical history to document cognitive decline and behavioural changes over time, often supplemented by input from family members. The NIA-AA criteria have become the most widely used in clinical and research settings, particularly with the introduction of the Amyloid, Tau, and Neurodegeneration (ATN) biomarker framework. The IWG criteria focus more on biomarker-based diagnosis, whereas the *DSM Fifth Edition* criteria provide broader classifications of neurocognitive disorders.

A variety of standardised cognitive tests are used in both clinical and research settings to assess cognitive function, including learning and memory, complex attention, executive function, language, perceptual and motor control, and social cognition [46]; the Mini-Mental State Examination (MMSE), Five-Word Test, Montreal Cognitive Assessment, digit span, clock drawing test, and logical memory tests I and II are some standard tests used to assess cognitive status [46–51]. While these tests provide valuable insight into cognitive impairment, they do not always correlate directly with biomarkers and imaging findings. For example, an individual may have amyloid and tau pathology but remain cognitively intact for years, making biomarker analysis essential for early detection. Furthermore, cognitive tests may have limitations due to cultural biases, educational background, and individual variability in cognitive reserve, which can influence test results. These cognitive tests are often supplemented by neuroimaging and biomarker analysis for a more precise diagnosis, such as Magnetic resonance imaging (MRI) to assess brain atrophy, PET scan to assess the levels of Aβ and tau, and CSF analysis to assess the levels of several biomarkers [52]. While neuroimaging is a valuable tool, its limitations include high costs, limited accessibility, and the need for specialised equipment. Additionally, MRI findings of atrophy are often non-specific and can overlap with other neurodegenerative disorders, making it necessary to integrate biomarker analysis for a more definitive diagnosis [53–56]. For research purposes, the NIA-AA research framework introduced the ATN classification in 2018, defining AD based on three key biomarker categories: amyloid (A), tau (T), and neurodegeneration (N). Amyloid pathology (A) is identified through low CSF Aβ_42_ levels, an abnormal Aβ_42_/Aβ_40_ ratio, and amyloid PET imaging. Tau pathology (T) is assessed through CSF phosphorylated tau (p-Tau 181) or tau PET imaging, while neurodegeneration (N) is measured using MRI-detected hippocampal atrophy, fluorodeoxyglucose PET for cerebral glucose metabolism, and CSF total tau levels [57]. In 2024, the ATN framework was updated to refine biomarker classification into core and non-core categories. The core biomarkers, which are specific to AD pathology, include core 1 biochemical markers such as CSF/plasma levels of Aβ_42_, p-Tau 217, p-Tau 181, p-Tau 231, and amyloid PET imaging and core 2 tau proteinopathy markers, including CSF levels of microtubule-binding tau fragments (Tau 243, p-Tau 205, and non-phosphorylated mid-region tau) and tau PET imaging. The non-core biomarkers relate to broader neurodegenerative processes and co-pathologies denoted by N. These include neurodegeneration (N), measured by CSF/plasma neurofilament light (NFL), anatomical MRI, and fluorodeoxyglucose PET; inflammation (I), assessed through mostly plasma GFAP levels, indicating astrocytic activation; vascular injury (V), detected via MRI-detected infarctions and white matter hyperintensities; and α-synuclein pathology (S), identified through CSF alpha-synuclein seed amplification assays, which help detect primary pathology or co-pathologies [19]. These refinements enhance the specificity of AD biomarkers, improving diagnostic precision and aiding in the early detection and differentiation of AD from other neurodegenerative conditions.

### Blood Biomarkers for AD

Blood biomarkers for AD offer a promising early detection and monitoring alternative that is minimally invasive and cost-effective, including Aβ_42_/Aβ_40_, amyloid precursor protein fragments, total Tau, and p-Tau [58]. They can be rapidly scaled up to meet the increasing need for biomarker testing, enabling widespread use in primary and secondary care and allowing for routine monitoring and clinical trial recruitment while reducing the healthcare burden [59, 60]. Although confirmatory PET or CSF testing may still be required, blood biomarkers improve accessibility and early intervention, making them a valuable tool for AD management [61].

Blood biomarkers for AD currently include Aβ_42_/Aβ_40_ ratio [62], hyperphosphorylated tau forms (p-Tau 181, p-Tau 217, p-Tau 231) [63, 64], and GFAP [22, 33, 65]. Studies have demonstrated a decreased plasma Aβ_42_/Aβ_40_ ratio correlated with AD brain pathology, though its diagnostic accuracy remains lower than CSF-based measurements [62, 66–69]. However, the accuracy of the plasma Aβ_42_/Aβ_40_ ratio as a diagnostic marker was lower in comparison to the CSF Aβ_42_/Aβ_40_ ratio [70]. Several physiological factors, such as enzymatic digestion, liver metabolism, and kidney function, may influence the amount of brain-derived Aβ in circulation [71–73]. Furthermore, plasma Aβ is not solely derived from the central nervous system (CNS) as peripheral tissues also contribute to its levels, potentially confounding diagnostic interpretations. These factors highlight the need for further refinement and standardisation to improve the clinical utility of blood-based AD biomarkers.

Elevated levels of plasma p-Tau 181 have been identified as a highly accurate biomarker for distinguishing AD from non-AD dementia [64, 74]. Similarly, plasma p-Tau 217 has demonstrated the ability to differentiate AD dementia from other neurodegenerative disorders and individuals who do not exhibit neuropathological features of AD [75–77]. More recently, plasma p-Tau 231 has emerged as an early biomarker capable of predicting the progression to AD and differentiating AD from other neurodegenerative conditions. Unlike p-Tau 181, plasma p-Tau 231 levels have been found to increase the entire Braak staging spectrum of AD, indicating its potential for detecting very early disease stages [63, 78]. However, plasma p-Tau 217 has been shown to outperform plasma p-Tau 181 due to its large dynamic range and higher fold difference between AD and healthy controls [79, 80].

Additionally, the activation of astrocytes—a process that appears to begin before symptoms manifest—has been identified as an early indicator of AD pathology [26, 27]. In particular, elevated plasma GFAP, a marker of astrocyte activation, has been associated with an increased risk of future AD dementia in preclinical stages [28, 29]. Plasma GFAP distinguished individuals with ongoing brain amyloid deposition (Aβ positive) from those with relatively low amyloid deposition (Aβ negative) compared to p-Tau 181, p-Tau 231, and NFL [30, 32, 81]. These findings highlight the potential of blood biomarkers for AD detection and monitoring disease progression. However, despite this promise, the performance of these assays varies widely. It lacks standardisation across laboratories, which raises the question of the minimum accuracy required for the blood test to be clinically useful. Furthermore, limiting the blood test to a single biomarker can lead to false positives or false negatives, impacting diagnostic accuracy.

## Function of Astrocytes and GFAP Expression in AD

### Role of Astrocytes in the CNS

Astrocytes comprise the most abundant glial cells in the brain, comprising 20–90% of total cells, depending on the brain area [82, 83]. Astrocytes regulate CNS function by facilitating neuronal communication at synapses, maintaining neurotransmitter balance, and coordinating calcium signalling through gliotransmitter release [23, 84, 85].

Along with endothelial cells and pericytes, astrocytic endfeet form an essential part of the blood–brain barrier. This barrier plays a crucial role in controlling the permeability of substances, maintaining cerebral blood flow, and regulating the composition of the extracellular fluid within the CNS [23, 24].

However, the exact role of astrocytes in neurodegenerative diseases remains highly debated. Some studies suggest that astrocytic dysfunction directly contributes to neurodegeneration, accelerating neuronal loss and disease progression [86–88]. In contrast, other research indicates that reactive astrocytes initially play a protective role, attempting to counteract early disease-related damage through neuroprotection and metabolic support [89, 90]. Thus, it remains unclear whether astrocytes predominantly drive pathology or serve as an initial defence mechanism in the early stages of neurodegeneration. This uncertainty highlights the need for further investigation into astrocyte subtypes, activation states, and their long-term impact on disease progression.

### Astrocyte Reactivity and Astrogliosis in Neurodegeneration

Under normal and pathological conditions, astrocytes exhibit a spectrum of molecular, cellular, and functional changes, transitioning into a reactive state known as astrogliosis [86–88]. Depending on the extent and nature of astrocyte activation, this response can be protective or harmful. Reactive astrocytes can support neuronal repair, maintain metabolic stability, and regulate immune responses. However, excessive activation may trigger inflammation, oxidative stress, and neurotoxicity, ultimately accelerating neurodegeneration. While some studies suggest that astrocyte reactivity serves as an early adaptive response to AD [89–91], others argue that chronic activation exacerbates disease progression [92, 93]. The extent to which reactive astrocytes contribute to neurodegeneration versus providing compensatory repair remains uncertain.

### GFAP Expression

GFAP is highly expressed in the cytoskeleton of astrocytes in the CNS. It is also present in the non-myelinating Schwann cells of the peripheral nervous system (PNS) and the glial cells of the enteric nervous system [94, 95]. While GFAP expression in the PNS is functionally similar to CNS astrocytes [94], aiding in structural support and nerve regeneration, GFAP in the enteric nervous system plays a distinct neuromodulatory role, influencing intestinal health, neuroimmune interactions, and gut-brain communication [96]. These functional differences may highlight how GFAP-expressing glial cells contribute to neurodegenerative and gastrointestinal disorders.

Overexpression of GFAP, along with other intermediate filaments such as vimentin and nestin, is a hallmark of astrocyte activation in response to neurological injury or disease [25]. These proteins play a crucial role in maintaining the structural integrity of astrocytes, helping to stabilise cellular structure and support neurological function [97–99]. However, while GFAP upregulation is often associated with a protective response to CNS damage, its presence in the bloodstream does not necessarily indicate a beneficial outcome. Instead, elevated plasma GFAP levels likely reflect astrocyte injury, neuroinflammation, or blood–brain barrier (BBB) dysfunction [100, 101].

Although GFAP is widely studied as a marker of astrocyte reactivity, whether its overexpression contributes to neuroprotection or brain pathology remains to be determined. Further research is needed to determine whether plasma GFAP elevation directly indicates disease severity or is simply a byproduct of CNS damage.

### Astrocyte Heterogeneity and GFAP Expression in AD

Astrocytes represent a heterogeneous population with diverse transcriptional, functional, and regional identities [102, 103]. Recent single-cell transcriptomic studies have revealed distinct astrocyte subtypes with specialised roles in synaptic regulation, neuroinflammation, and metabolic support across various CNS disorders [103]. In AD, multiple transcriptionally distinct astrocyte subpopulations with diverse roles have been identified that exhibit region-specific, functional, and pathological differences [104, 105]. Certain subtypes may be selectively vulnerable or protective in the disease process, and their depletion or expansion could contribute to the heterogeneity seen in GFAP expression and release. Astrocytes with high GFAP expression, including disease-associated astrocytes, have been mostly reported to expand in AD and play a role in neuroinflammation, metabolic dysregulation, and blood–brain barrier dysfunction [103, 106, 107]. The identification of a specific diminished astrocyte subpopulation in AD, characterised by low GFAP expression and high expression of AQP4 and CD63, proteins associated with Aβ clearance and tau protein binding, suggests a loss of protective astrocytic functions in disease progression [108]. Their selective dysfunction or expansion could contribute to increased GFAP release into interstitial fluid and plasma, particularly under conditions of blood–brain barrier compromise or glymphatic dysfunction [109, 110]. These subtypes may represent key GFAP reservoirs, and their activation state could disproportionately influence GFAP dynamics in biofluids, adding further complexity to biomarker interpretation. These insights emphasise that the plasma GFAP elevations may reflect the selective loss or expansion of particular astrocyte subsets rather than a uniform activation state. A better understanding of GFAP expression across astrocyte subpopulations is critical for interpreting GFAP dynamics in AD and enhances its utility as a fluid biomarker for capturing specific astrocytic responses.

### GFAP as a Biomarker in Neurological Disorders

Elevated GFAP levels in CSF and plasma have been observed across multiple neurological conditions, including AD, Parkinson’s disease, Huntington’s disease, traumatic brain injury (TBI), stroke, multiple sclerosis, neuromyelitis optica spectrum disorder, cerebral amyloid angiopathy, and prion disease [111–119]. Plasma GFAP elevations have also been reported in postoperative delirium [120], although findings across studies are inconsistent [121–123]

Under these conditions, astrocytes may undergo structural and functional changes, releasing GFAP into the interstitial and extracellular fluids [124]. While the BBB, blood-CSF, and arachnoid barriers generally restrict the movement of large proteins [125, 126], neuroinflammation or CNS injury can compromise these barriers, allowing GFAP to enter circulation [127–129]. The glymphatic system also plays a role in GFAP transport from interstitial fluid to CSF and eventually into the bloodstream [130, 131]. Currently, the U.S. Food and Drug Administration has approved blood GFAP testing for moderate traumatic brain injury, specifically to assess the need for a CT scan within 12 h of injury [115, 116, 132–135]. Despite promising findings, plasma GFAP is not yet validated as a reliable biomarker for AD diagnosis, as its elevation is observed in multiple neurological disorders. Therefore, whether astrocytosis and elevated GFAP represent simply a secondary response to neuronal injury or an active participant in the disease process remains unclear. Further studies are necessary to distinguish its role in AD pathology from other neurological conditions and determine its potential for monitoring disease progression.

### Astrocyte Response to Aβ in AD

Reactive astrocytes have been identified near amyloid plaques in early-stage AD, suggesting that Aβ deposition triggers astrocyte activation [136]. This activation stimulates the release of proteases, including neprilysin, endothelin-converting enzyme, insulin-degrading enzyme, and matrix metalloproteases, which hydrolyse Aβ, potentially limiting plaque formation [137]. Additionally, astrocytes secrete extracellular protein chaperones, such as apolipoprotein E (APOE), apolipoprotein J, α2-macroglobulin, and α1-antichymotrypsin, which aid in Aβ clearance across the BBB and via the glymphatic system [137, 138]. While these mechanisms highlight the potential neuroprotective role of astrocytes in AD, conflicting evidence suggests that as Aβ deposition progresses, astrocytic functions become impaired, leading to inefficient Aβ clearance and increased neuronal toxicity. The precise role of reactive astrocytes in Aβ accumulation and degradation remains unclear.

### GFAP and Astrocyte Dysfunction in AD Progression

In preclinical AD, increased plasma GFAP levels have been linked to higher cerebral glucose consumption, suggesting that reactive astrocytes become metabolically active in response to early Aβ pathology [139]. However, as Aβ deposition advances, astrocytes undergo functional impairments, disrupting key regulatory mechanisms and promoting neurodegeneration. One major consequence of this dysfunction is Ca^2+^ signalling dysregulation, which alters gliotransmitter release and astrocyte-neuron communication [140]. Additionally, reactive astrocytes contribute to neuroinflammation, releasing cytokines, nitric oxide, and reactive oxygen species, further amplifying oxidative stress and neuronal damage [141, 142]. This chronic dysfunction leads to glutamatergic excitotoxicity [143], impaired Aβ clearance [138], abnormal glucose metabolism [144], and increased production of proinflammatory cytokines [145], all contributing to AD progression. Although plasma GFAP elevation reflects astrocyte activation, the biological significance of this response remains uncertain. Understanding these mechanisms is crucial for identifying potential therapeutic targets to prevent or slow neurodegeneration in AD. The astrocyte function in the normal stage, the reactive stage in early AD, and astrocyte dysfunction in AD progression are summarised in Table 1..

**Table 1.** Astrocyte function in the normal stage, reactive astrocytes and astrocyte dysfunction in AD progression

Astrocyte functions	Normal astrocytes	Reactive astrocytes in AD	Astrocytes dysfunction in AD
Neurotransmitter balance and synaptic regulation	Maintain neurotransmitter homeostasis and support synaptic communication [23]	-	-
Calcium signalling and gliotransmitter release	Coordinate neuronal signalling through gliotransmitter release [84, 85]	-	-
BBB maintenance	Form part of the BBB, regulating permeability and extracellular fluid composition [23, 24]	-	-
Neuroprotection and metabolic support	Support neuronal function by maintaining metabolic stability [23, 84, 85]	React to Aβ deposition by releasing neuroprotective factors [89–91]	-
Aβ clearance through proteases and chaperones	Contribute to Aβ clearance mechanisms [137, 138]	Increase Aβ hydrolysis via proteases and extracellular chaperones[137, 138]	-
GFAP overexpression	-	Upregulate GFAP and other intermediate filaments [25, 97–99]	-
Increased metabolic activity	-	Show increased GFAP levels linked to higher cerebral glucose consumption [139]	-
Ca^2+^ signalling dysregulation and neuroinflammation	-	-	Exhibit Ca^2+^ signalling disruptions, impairing gliotransmitter release and neuron communication [140]
Impaired Aβ clearance and glutamatergic excitotoxicity	-	-	Fail to effectively clear Aβ, leading to increased neurotoxicity [138, 143]
Oxidative stress and neuronal toxicity	-	-	Promote neuroinflammation, oxidative stress, and excitotoxicity, exacerbating neurodegeneration [141–145]

*AD* Alzheimer’s disease, *Aβ* amyloid beta, *BBB* blood–brain barrier, *GFAP* glial fibrillary acidic protein

## GFAP Proteome

**Fig. 1:**
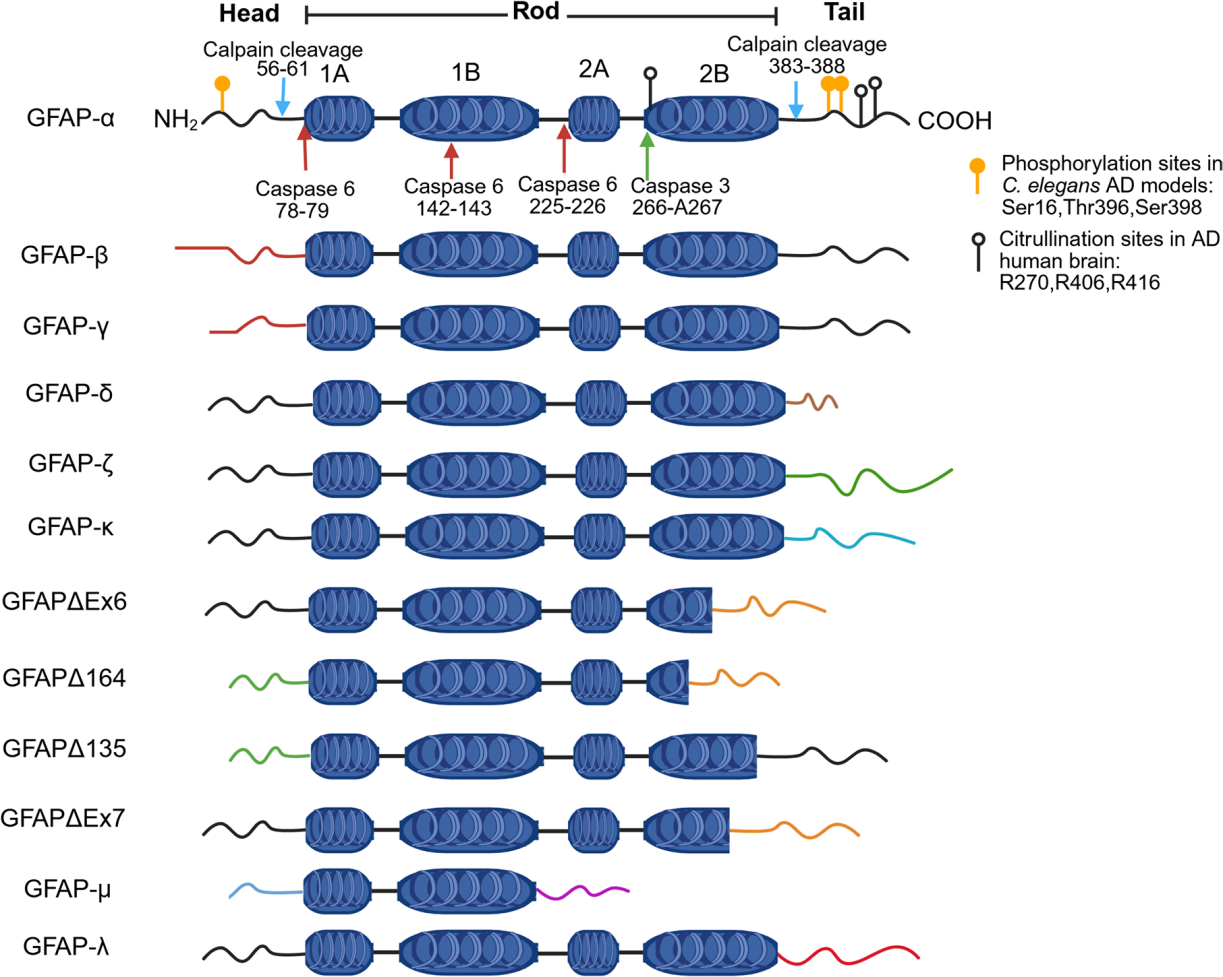
GFAP linear structure, functional domain, and isoforms (different colours of N and C terminal indicate alterations in domains) (created with BioRender.com); AD, Alzheimer’s disease; GFAP, glial fibrillary acidic protein

GFAP is an intermediate filament III protein (8–12 nm) found as a key component in the cytoskeleton of astrocytes. It plays a crucial role in maintaining the structural integrity of the cell [132]. GFAP was initially identified by Eng et al. [146] in severe fibrous gliosis tissues of old multiple sclerosis plaques from an individual with hydrocephalus ex vacuo. GFAP filaments possess a central α-helical rod domain flanked by variable head and tail domains, showing structural similarities with other intermediate filaments (vimentin, desmin, and peripherin). The predominant form of GFAP-α is composed of four coils (1A, 1B, 2 A, 2B), linked by three linker regions, forming the rod domain, which is a highly conserved structural organisation in class III intermediate filament proteins (Fig. 1) [132]. The transition of GFAP monomers to the filamentous form involves assembly into parallel dimers, followed by antiparallel binding to form tetramers up to 10 nm thick. The N-terminal head domain is essential for filament elongation, while the C-terminal tail domain facilitates oligomerisation [147, 148].

### GFAP Isoforms

Despite being encoded by a single gene on chromosome 17q21, GFAP exists in multiple isoforms, generated through C-terminal variants, alternative splicing, and alternative start sites, contributing to its functional diversity across different nervous system compartments [149]. Twelve GFAP isoform transcripts have been identified in the human brain, with a similar profile observed in the mouse brain (Fig. 1) [150].

Understanding these isoforms is crucial, as they exhibit distinct structural features, tissue distribution, and functional implications in normal physiological processes and neurodegenerative conditions, including AD. However, the precise functional roles of these isoforms remain largely unexplored, and the extent to which they contribute to pathological mechanisms or protective responses in neurodegeneration is still debated.

#### Canonical, N-Terminal, and C-Terminal GFAP Isoforms

GFAP-α is the most abundant isoform, forming the primary component of astrocyte intermediate filaments [151]. This isoform, which comprises nine exons and 432 amino acid residues, is highly expressed in the brain and spinal cord astrocytes, with lower levels detected in the PNS [151].

GFAP-β and GFAP-γ arise from alternative transcriptional start sites within the GFAP gene, resulting in distinct N-terminal sequences and an identical C-terminal to GFAP-α. GFAP-β originates from an alternative site upstream of intron 1 and is primarily expressed in the PNS, Schwann cells, hepatic stellate cells, and brain [152, 153]. It has also been detected in gliomas and lymphocytes, suggesting a potential role in tumour biology. GFAP-γ, which starts within exon 4, produces a truncated protein lacking the head and part of the rod domains, potentially influencing astrocyte motility and cytoskeletal interactions [154]. GFAP-γ is enriched in the mouse brain, bone marrow, spleen, and human brain [154].

GFAP-ε/δ is predominantly expressed in neurogenic astrocytes within the subventricular zone and subpial astrocytes [155, 156], featuring an alternative exon (7a) and lacking exons 8 and 9, resulting in a unique C-terminal sequence [157]. GFAP-κ, found in enteric glial cells, contains a different exon 7b, distinct from both GFAP-α and GFAP-ε/δ, while sharing the same head and rod domains [158]. GFAP-ζ is another isoform characterised by an altered C-terminal region, potentially due to including nucleotides in the last intronic sequences in exon 9 [159, 160]. GFAP λ is another splice variant isoform that is overexpressed in patients with Type-II Alexander Disease, resulting in the formation of Rosenthal fibres and the destruction of normal astrocyte function. This isoform includes an additional exon, termed exon 7 C, which is inserted between exons 7 and 8, producing a longer transcript than the predominant GFAP-α isoform [161].

The novel GFAP isoform, termed GFAP µ, was identified in glioma tissue and healthy brain. This isoform is formed by alternative splicing where exon 2 is skipped, leading to a premature termination codon in exon 3 with an altered C-terminus compared to GFAP-α [162].

#### Frame-Shift (Out-of-Frame and In-Frame) Variants

Alternative splicing generates multiple GFAP variants with distinct structural properties and functional implications [159, 163]. Splice variants, including GFAPΔEx6, GFAPΔ164, and GFAPΔEx7, encode for a wild-type N terminus and out-of-frame C-terminal sequence, leading to the GFAP + 1 protein, which is expressed in a subset of astrocytes. GFAP + 1 refers to a specific protein that is a product of alternative splicing of the GFAP gene, specifically resulting from the GFAPΔEx6, GFAPΔ164, and GFAPΔEx7 out-of-frame splice forms [159, 163, 164]. GFAPΔ164, which has shortened exons 6 and 7, and GFAPΔexon6, which lacks exon 6, are devoid of the coil 2B region. GFAPΔEx7 is another out-of-frame splice variant lacking exon 7 [132, 150]. Due to the frameshift alteration, these isoforms have different C-terminal regions of GFAP-α. Another splice variant, GFAPΔ135 is an in-frame splice variant that features a 135-nucleotide deletion in exon 6, resulting in coil 2B region loss while retaining the same C-terminal sequence as GFAP-α [132].

The summary of the location and structural differences between GFAP isoforms compared to canonical GFAP-α is stated in Table 2..

**Table 2.** Summary of GFAP isoforms: locations, structural differences compared to GFAP-α, and expression in AD

GFAP isoform	Transcriptional differences with GFAP-α	Location in the human body	mRNA and protein expression in AD samples	Reference
GFAP-α (canonical isoform)	Not applicable	Astrocytes in the brain and spinal cord, to a lesser extent in PNS	Both mRNA and protein are expressed and increased in astrocytes near Aβ plaques	[150, 164]
N-terminal variants				
GFAP-β	Different N-terminal region from alternative start site upstream of intron 1. Identical C-terminal to GFAP-α	Gliomas, lymphocytes and human brain	mRNA expression in the brain hippocampus of AD	[150, 153, 165]
GFAP-γ	Different N-terminal region from alternative start site upstream of intron 1, mRNA excludes exon 1 and includes the last ∼130 nt of intron 1, identical C-terminal to GFAP-α	Human brain	Not reported	[154]
C-terminal Variants				
GFAP-δ (GFAP-ε/δ)	Contains an alternative exon (exon 7a) within intron 7, lack of exon 8 and 9, a different C-terminal region	Neurogenic astrocytes in the subventricular zone and subpial astrocytes	Both mRNA and protein are expressed and increased in astrocytes near Aβ plaques. The binding capacity of GFAP-δ to PS1 is high	[150, 155, 156, 166]
GFAP-ζ	Contains the last intronic nucleotide sequence in exon 9, a different C-terminal region	mRNA expressed in the human brain	mRNA expressed in both control and AD brain	[150]
GFAP-κ	Contains a different exon 7 (exon 7b), different C-terminal region	Human enteric cells	mRNA expressed in both control and AD brain	[150, 167]
GFAP λ	Additional exon, termed exon 7 C, which is inserted between exons 7 and 8, is a different transcript than the GFAP-α isoform	Expressed in the healthy human brain but overexpressed in Alexander disease	Not reported	[161]
N and C-terminal variant				
GFAP μ	Alternative splicing where exon 2 is skipped, leading to a premature termination codon in exon 3	Human healthy brain	Not reported	[162]
Frame-shift (out-of-frame) C-terminal variants				
GFAPΔEx6	Lacks exon 6, wild type N-terminus, out-of-frame C-terminus	A specific subpopulation of astrocytes in the human hippocampus	Isoforms detected with mRNA expression. Protein expression was validated from GFAP + 1 antibody, which can identify GFAPΔEx6, GFAPΔ164 and GFAPΔEx7 out-of-frame proteins	[163, 164]
GFAPΔ164	Shortened exons 6 and 7, lacks coil 2B region, remain wild type N-terminus and out-of-frame C-terminus	A specific subpopulation of astrocytes in the human hippocampus	Isoforms detected with mRNA Expression. Protein expression was validated from the GFAP + 1 antibody, which can identify GFAPΔEx6, GFAPΔ164 and GFAPΔEx7 out-of-frame proteins	[163, 164]
GFAPΔEx7	Lacks exon 7, wild type N-terminus, out-of-frame C-terminus	A specific subpopulation of astrocytes in the human hippocampus	mRNA expressed in both control and AD brain	[150]
Frame-shift (in-frame) isoform with canonical C-terminal				
GFAPΔ135	Deletion of 135 nucleotides in exon 6 lacks coil 2B region, identical C-terminal to GFAP-α	A specific subpopulation of astrocytes in the human hippocampus	Isoforms detected with mRNA Expression. Protein expression was validated from GFAPpan antibodies, which can identify both GFAP-α and GFAPΔ135	[150, 163]

*AD* Alzheimer’s disease, *GFAP* glial fibrillary acidic protein, *PNS* peripheral nervous system

### Functional Impact of GFAP Isoforms on Astrocyte Behaviour

Different GFAP isoforms contribute uniquely to cytoskeletal regulation, affecting astrocyte motility, cellular interactions, and neuroinflammatory responses [168]. Variations in the C-terminal region alter binding properties with interacting proteins, influencing astrocyte migration [155, 166, 169], wound healing, and response to brain injury [166, 170, 171]. GFAP-α provides cytoskeletal stability, ensuring structural resilience, while GFAP-ε/δ and GFAP-κ, with altered C-terminal sequences, may modulate astrocyte plasticity and regenerative potential.

Different GFAP isoforms influence cytoskeletal organisation, which may explain why some astrocytes remain functional while others become reactive or dysfunctional in AD [156, 159] and other neurodegenerative conditions [161]. However, the mechanisms through which specific GFAP isoforms contribute to disease progression remain unclear. Some isoforms may enhance neuronal protection, while others could drive reactive gliosis and neuroinflammation.

Advanced single-cell RNA sequencing and proteomic studies are needed to map their expression in different astrocyte subtypes and investigate their interaction with neuroinflammatory pathways, potentially identifying new therapeutic targets.

### GFAP Isoforms in AD

#### GFAP Expression and Astrocyte Activation in Early AD

In the early stages of AD, amyloid plaques are surrounded by activated astrocytes [136, 159, 172], increasing GFAP expression, which correlates with disease severity [28, 173]. Several GFAP isoforms, including GFAP-α, GFAP-δ, GFAP-ζ, GFAP-κ, GFAPΔEx7, and GFAPΔ135, have been significantly upregulated in the hippocampus [150, 156]. This suggests that these GFAP isoforms may play a role in the disease, either as a response to brain changes or as contributors to the progression of AD. Additionally, transcript levels of GFAPΔEx6 and GFAPΔ164 have been detected in both cognitively unimpaired individuals and AD patients, suggesting that certain GFAP isoforms are expressed before the onset of clinical symptoms [150].

Immunostaining studies have shown that GFAP-α, GFAP-δ, and spliced isoforms such as GFAPΔEx6, GFAPΔ164, and GFAPΔ135 are expressed in the human hippocampus [156, 164]. Among the identified GFAP splice variants, GFAPΔEx6 and GFAPΔ164 have been linked to the expression of GFAP + 1 protein, a marker of a unique subset of astrocytes. Unlike typical reactive astrocytes, these GFAP + 1-expressing astrocytes do not exhibit typical reactive astrocyte characteristics. The expression of GFAP + 1 increases as AD progresses, suggesting a potential role in disease advancement. However, whether these GFAP + 1-positive astrocytes contribute to neuroprotection or neurodegeneration remains unknown [150]. Notably, increased GFAP-α and GFAP-δ expression has been observed around amyloid plaques in the AD hippocampus, highlighting their potential involvement in plaque-associated astrocyte reactivity [150, 156]. GFAP-δ is particularly enriched in the proximal processes of astrocytes and is widely distributed throughout the hippocampal region [150]. However, the exact role of GFAP-δ in astrocytic function and its broader implications in AD pathology remain unclear.

#### Functional Interaction Between GFAP-δ and Presenilin Proteins in AD

The functional significance of GFAP-δ in AD has been suggested through its interaction with presenilin protein (PS1), a key component of the γ-secretase complex involved in amyloid precursor protein cleavage [174, 175]. Studies have shown that GFAP-δ has a higher binding capacity to PS1 than GFAP-α in yeast and in vitro, which may influence amyloid precursor protein processing and amyloid plaque formation [166]. The increased expression of GFAP-δ and PS1 in reactive astrocytes surrounding senile plaques further supports the idea that GFAP-δ may play a critical role in AD pathology [174]. The summary of different GFAP isoforms and their expression in AD is listed in Table 2..

Despite the growing evidence of GFAP isoform involvement in AD, several knowledge gaps remain. The functional significance of increased GFAP-δ expression around amyloid plaques and its interaction with PS1 remains unclear. Additionally, while the presence of GFAP + 1-expressing astrocytes has been confirmed, their exact role in disease progression and neuronal survival is yet to be determined. Future studies should focus on understanding whether GFAP isoform expression reflects a compensatory mechanism to maintain astrocyte function or contributes to neurodegeneration. Investigating the molecular pathways influenced by GFAP isoforms could provide novel insights into potential therapeutic targets for AD.

### Post-Translational Modifications of GFAP in Neurodegeneration

GFAP undergoes various post-translational modifications (PTMs), such as phosphorylation, citrullination, acetylation, and others, which play crucial roles in regulating its function, assembly, and interactions [176, 177]. GFAP post-translational modifications regulate astrocyte function and structural stability, but when dysregulated, they can lead to astrocyte dysfunction and neuroinflammation [135, 178, 179]. Excessive phosphorylation disrupts GFAP polymerisation, while abnormal citrullination and acetylation weaken the cytoskeleton, altering astrocyte behaviour. These changes can trigger astrocyte reactivity, increase inflammation, and contribute to neurodegenerative diseases like Alzheimer’s [180, 181].

#### Phosphorylation of GFAP and Its Functional Implications

Phosphorylation is one of the most common PTMs of GFAP, primarily occurring in the N-terminal domain (Thr7, Ser8, Ser13, Ser17, Ser34/38, Ser59) and, to a lesser extent, in the C-terminal domain (Thr383, Ser398). These phosphorylation events are regulated by protein kinase A, calmodulin-dependent protein kinase II, and protein kinase C. The high concentration of phosphorylation sites in the N-terminal domain inhibits GFAP polymerisation and filament assembly [182], affecting astrocyte structural stability. Increased expression of phosphorylated GFAP at specific sites (Ser8 and Ser13) has been observed in hypoxic brain injury in neonatal pig brains. At the same time, Ser13 phosphorylation has been associated with aggressive infantile Alexander disease and frontotemporal dementia [135, 178, 179]. In *C. elegans* AD models, additional phosphorylation sites (Ser16, Ser398 and Thr396) were identified in the hippocampus, particularly in APOEε4 carriers compared to APOEε3 models (Fig. 1) [183].

Phosphorylation plays an important role in maintaining the equilibrium between polymerised and depolymerised subunits of GFAP [178], thereby regulating astrocyte motility, mitotic remodelling, and plasticity. Phosphorylation has been linked to G protein–coupled mGluR receptor-induced calcium influx, which influences synaptic plasticity and astrocytic interactions with neurons [176, 177]. Further regulation of GFAP assembly contributes to the extensive remodelling of glial frameworks during mitosis [182]. However, excessive phosphorylation can destabilise the astrocyte cytoskeleton, disrupting the structural integrity of astrocytes and impairing their ability to regulate the extracellular environment. This may lead to altered calcium influx, excessive glutamate accumulation, and neuroinflammation, potentially exacerbating excitotoxic cell death and neuronal degeneration [178].

#### Citrullination of GFAP and Its Role in Neuroinflammation

Citrullination is another post-translational modification observed in GFAP, where arginine residues (R30, R36, R270, R406, and R416) are converted to citrulline by peptidyl arginine deiminases (PAD) in a calcium-dependent manner [184]. While the full extent of citrullinated GFAP’s effects remains unclear, it has been implicated in autoimmune responses related to neurological diseases, interference with GFAP polymerisation, and disease-specific variations in citrullination levels [180, 185–187]. An abnormal accumulation of citrullinated GFAP, mainly in R270, R406, and R416 sites (Fig. 1), was found in hippocampal brain samples of AD patients, demonstrating its significance as a biomarker of neurodegeneration [180, 181]. PAD2, the enzyme responsible for GFAP citrullination, is significantly upregulated in hippocampal extracts from AD patients, suggesting a link between increased PAD2 activity and AD pathology [181]. The presence of citrullinated GFAP has also been observed in multiple sclerosis [185, 186], hepatic fibrosis [187], and other neuroinflammatory disorders, raising questions about whether citrullination is a general marker of neuroinflammation or plays a unique role in AD progression.

#### Acetylation of GFAP and Its Influence on Cytoskeletal Dynamics

Acetylation is another modification that occurs on GFAP, affecting multiple lysine residues across its structure. This modification is mediated by acetyltransferases, which transfer acetyl groups to lysine residues using acetyl-CoA as a donor molecule [188]. Histone acetylation has been shown to inhibit GFAP expression and reorganise the IF network, altering astrocyte function [189]. In amyotrophic lateral sclerosis patients, differential GFAP acetylation was observed at K89, K153, K189, K218, K259, and K331 sites of GFAP in the spinal cord. Although acetylation modifications are widespread across the GFAP protein, their effects on GFAP structure and functions and which sites are acetylated in AD are currently unknown and warrant further investigation [190].

#### Implications of GFAP PTMs on Immunoassay Detection in AD

One critical issue of GFAP PTMs is their potential to interfere with antibody binding in immunoassays used for clinical diagnostics [191]. Since PTMs can modify GFAP structure, they may alter epitope availability, accuracy, and sensitivity of GFAP-based biomarker assays. Citrullination and acetylation, in particular, could mask key binding sites, while phosphorylation-induced conformational changes might affect antibody-paired assays [192]. Given the increasing interest in using GFAP as a biomarker for AD, it is essential to characterise the impact of PTMs on the analytical performance of GFAP immunoassays to ensure reliable detection in clinical applications. Although significant progress has been made in understanding GFAP post-translational modifications, several knowledge gaps remain. The exact role of GFAP phosphorylation in astrocyte dysfunction and neurodegeneration is still unclear, and it is unknown whether targeting specific phosphorylation sites could be beneficial or detrimental in AD therapy. Similarly, the contribution of citrullinated GFAP to neuroinflammation and disease progression remains uncertain, and further studies are required to determine whether PAD2 inhibition could serve as a viable therapeutic approach.

Additionally, GFAP acetylation patterns in AD remain primarily uncharacterised, leaving open questions about their influence on astrocyte behaviour and disease progression. Future studies should focus on using mass spectrometry-based proteomics and advanced imaging techniques to map site-specific GFAP PTMs in AD brains. Understanding how these modifications influence GFAP function, astrocyte behaviour, and neuroinflammatory responses could provide critical insights into their role in disease progression. Additionally, evaluating the impact of PTMs on GFAP biomarker detection in clinical assays is necessary to enhance the reliability and accuracy of GFAP-based diagnostics for neurodegenerative diseases.

### GFAP Proteolysis and Fragmentation in Neurodegeneration

GFAP undergoes proteolysis or fragmentation, producing GFAP breakdown products (GFAP-BDPs) that may influence cellular stability and neurodegeneration. In vitro and in vivo fragmentation processes are mediated primarily by calcium-activated proteases like calpain and caspases [193, 194]. The resulting truncated GFAP fragments range from 38 to 44 kDa, compared to the full-length 50-kDa protein [194, 195]. While GFAP fragmentation plays a physiological role in astrocyte remodelling, its dysregulation has been linked to neurodegenerative diseases such as AD [196, 197], amyotrophic lateral sclerosis [198], and TBI [132, 194]. However, the full impact of GFAP fragmentation in chronic neurodegeneration, particularly in AD, remains poorly understood.

#### Calpain- and Caspase-Mediated GFAP Fragmentation

Calpain, a calcium-activated protease, is a major regulator of GFAP cleavage at both the N- and C-terminal domains, producing fragments that interfere with GFAP filament assembly. The main calpain cleavage sites in GFAP are N56-A61 (N-terminal) and T383-F388 (C-terminal) (Fig. 1) [199–201]. This truncation preserves the rod domain, which is essential for filament formation, but removes the head and tail domains, preventing proper filament assembly. In neurodegenerative diseases such as amyotrophic lateral sclerosis, TBI, and post-mortem human brain tissue, calpain-mediated fragmentation is a common feature [132, 194, 198]. In experimental and human TBI cases, calpain-mediated GFAP-BDPs (44–38 kDa) dominate, suggesting that acute injury results in a distinct GFAP fragmentation profile. Additionally, GFAP-BDPs (38 kDa) have been shown to outperform full-length GFAP in distinguishing TBI from healthy individuals, emphasising its potential as a biomarker for acute brain injury [193]. However, whether calpain-mediated GFAP fragmentation plays a similar role in chronic neurodegenerative diseases like AD is still unknown.

In addition to calpain, caspases—particularly caspases 3 and 6—can cleave GFAP at various sites. Caspase 6 cleaves GFAP at epitopes 78/79, 142/143 and 225/226, particularly affecting the coil 1 A and linker regions before coil 2 A (Fig. 1) [132, 193, 202, 203]. In AD, caspase 3 has been implicated in GFAP fragmentation, producing a distinct 20-kDa N-terminal fragment at site 266-A267, located at the start of coil 2B (Fig. 1) [204]. This suggests that different fragmentation pathways may dominate specific diseases, with caspase-mediated cleavage playing a more significant role in chronic neurodegeneration such as AD.

#### Potential Implications for Diagnosis and Biomarker Development

The differential fragmentation patterns of GFAP could provide diagnostic value in distinguishing acute from chronic neurodegenerative conditions. In TBI, calpain-mediated GFAP-BDPs are the primary fragments detected in CSF, and measuring both total GFAP and its breakdown products has improved TBI diagnostics [193]. However, the role of GFAP-BDPs in AD and their presence in biofluids remain largely unexplored. Since GFAP fragmentation affects filament stability and astrocyte function, understanding the balance between physiological and pathological GFAP cleavage is critical. Whether GFAP-BDPs contribute to AD pathology or are simply byproducts of neurodegeneration remains unclear. Future research should investigate whether fragmented GFAP in AD biofluids correlates with disease progression or cognitive decline, which could establish GFAP-BDPs as biomarkers for astrocyte dysfunction in AD.

## Blood and CSF GFAP Levels in AD

GFAP has been increasingly recognised as a potential biomarker for AD, with elevated levels observed in plasma and CSF. However, differences in the clinical utility and stability of GFAP in blood and CSF suggest that plasma GFAP may be a more reliable biomarker for early AD detection. While plasma GFAP levels correlate strongly with Aβ pathology and cognitive decline, their relationship with tau pathology remains unclear, raising questions about whether GFAP elevation occurs independently of neurodegeneration or as part of a compensatory astrocytic response.

### Plasma GFAP as an Early Indicator of AD Pathology

Elevated plasma GFAP levels have been observed in cognitively unimpaired individuals, those with mild cognitive impairment (MCI), and AD patients, particularly in cases with Aβ accumulation detected by PET imaging [22, 205–208]. These findings suggest that plasma GFAP may serve as a predictive biomarker for AD progression, particularly for identifying individuals at risk of converting from MCI to AD [22, 28, 209, 210]. Increased plasma GFAP levels have also been associated with accelerated cognitive decline across different stages of disease severity, supporting its role in monitoring disease progression [28, 33, 173, 205, 211, 212]. Notably, plasma GFAP levels are elevated as early as 10 years before the onset of cognitive symptoms in individuals who later developed AD [213]. Furthermore, elevated plasma GFAP levels were significantly associated with faster cognitive decline among APOE ε4 carriers, suggesting a high vulnerability of astrocytosis and neuroinflammation in genetically high-risk groups [214]. Supporting these findings, Phillips et al. [215] demonstrated that higher GFAP gene and protein expression in cortical brain regions was significantly associated with higher brain amyloid burden and faster cognitive decline. This observation aligns with plasma biomarker studies, highlighting that elevated plasma GFAP levels reflect astrocyte activation in response to AD pathology.

Interestingly, while plasma GFAP levels correlate strongly with Aβ pathology, studies indicate no direct association with tau pathology [205, 208, 209, 216]. Some studies suggest that GFAP elevation might occur independently of Aβ pathology and neurodegeneration [173], possibly reflecting early astrocytic dysfunction rather than direct neuronal damage. Additionally, increased plasma GFAP has been linked to structural brain changes, including decreased cortical thickness, increased white matter hyperintensities, and a higher incidence of cerebral microbleeds in MCI patients [27, 29, 212]. These structural alterations further highlight the potential utility of plasma GFAP as a marker of neuroinflammatory processes in AD.

### Longitudinal Studies Supporting Plasma GFAP as a Predictive Biomarker

Several longitudinal biomarker studies confirmed the reliability of plasma GFAP for AD risk assessment. In a study tracking cognitively unimpaired older adults over 12 months, plasma GFAP levels combined with other AD risk factors (age, sex, and APOE ε4 status) showed the highest accuracy in predicting brain amyloidosis compared to other blood-based biomarkers such as total-Tau, p-Tau 181, p-Tau 231, and NFL [32]. Plasma GFAP also demonstrated the strongest effect size in distinguishing Aβ-positive from Aβ-negative individuals, reinforcing its value as a preclinical AD biomarker [32].

Similarly, 17-year community-based cohort studies identified plasma GFAP levels as a robust indicator (compared to p-Tau 181 and NFL) for predicting AD risk, with levels remaining significantly elevated in AD participants over time [30, 81]. Another 16-year longitudinal study linked higher serum GFAP levels with sporadic AD development, cognitive decline, and structural brain changes, emphasising its potential for tracking disease progression [217].

Most recently, Varma et al. [213] reported that plasma GFAP levels were elevated at least 10 years prior to the onset of cognitive symptoms in AD, with levels strongly associated with AD neuro pathology. The study highlights plasma GFAP as an early marker of reactive astrocytosis and AD pathogenesis. Additionally, Abbas et al. [218] demonstrated that the longitudinal increase of plasma GFAP is significantly associated with cognitive decline and supports the potential use of plasma GFAP as a secondary endpoint in AD clinical trials.

Notably, in patients with Down’s syndrome associated with AD, GFAP levels begin to increase around the age of 40, mirroring the neuropathological progression seen in both autosomal and sporadic AD [18, 219]. These findings suggest that plasma GFAP elevation occurs more than a decade before clinical diagnosis, highlighting its potential for early risk assessment and intervention strategies. Table 3. comprehensively summarises studies on blood GFAP levels in AD.

**Table 3.** Key studies of blood GFAP levels in AD

Author, year, and country (cohort)	Method, Sample type/storage	AD diagnosis criteria	Participants and covariates	Type of assay	Analytical validation	Major outcome
**GFAP as an early blood biomarker in AD**						
**Stocker et al. (2023) **[30]**,** **Germany**	Prospective cohort (17 years), lithium-heparin plasma, − 80 °C	NIA-AA or IWG-2	AD, 145 Vascular dementia, 66 Mixed dementia, 50 Control, 507 Covariates: age, sex, and APOE ɛ4 status	Neurology 4-Plex E Advantage kits (Quanterix, Billerica, MA, USA)	High and low QC	GFAP was associated with clinical AD incidence even more than a decade before diagnosis (9–17 years)
**Chatterjee et al. (2021) **[22]**,** **Australia (KARVIAH cohort)**	Cross-sectional, fasting EDTA plasma, − 80 °C	Aβ PET: Aβ positive-SUVR ≥ 1.35 Aβ negative-SUVR < 1.35 and MMSE score	Aβ positive, 33 Aβ negative, 63 Covariates: age, sex, and APOE ɛ4 status	SIMOA GFAP Discovery kits (Quanterix, Billerica, MA, USA)	3-level QC Repeatability, 0–14 CV% Reproducibility, 8–16 CV%	Plasma GFAP levels are increased in cognitively normal older adults with high brain Aβ load
**Benedet et al. (2021) **[208]**,** **Canada (TRIAD cohort),** **Spain (ALFA + study), France (BioCogBank Paris Lariboisière cohort)**	Cross-sectional, plasma, − 80 °C	Aβ-PET, tau-PET, CSF Aβ_42_/Aβ_40_	TRIAD cohort: *n* = 300 (young CU, *n* = 35; CU/Aβ negative, *n* = 114; CU/Aβ positive, *n* = 42; MCI, *n* = 39; AD, *n* = 45; non-AD, *n* = 25) ALFA + cohort: *n* = 384 (CU/Aβ negative, *n* = 249; CU/Aβ positive, *n* = 135) BioCogBank Paris Lariboisière: *n* = 187 (CU/Aβ negative, *n* = 21; MCI, *n* = 42; AD, *n* = 76; non-AD, *n* = 48) Covariates: age and sex	SIMOA GFAP Discovery kits (Quanterix, Billerica, MA, USA)	NA	Plasma GFAP levels were significantly higher among individuals with preclinical AD and reached their higher levels at symptomatic stages of AD
**Prins et al. (2022) **[206]**,** **The Netherlands**	Cross-sectional, EDTA plasma, − 80 °C	Aβ positive: CSF Aβ_42_ < 1000 pg/mL, p-Tau/Aβ_42_ ratio > 0.02	HC, 50 Pre-clinical AD, 50	SIMOA GFAP Discovery kits (Quanterix, Billerica, MA, USA)	NA	GFAP was significantly higher in subjects with preclinical AD compared to healthy elderly
**Pereira et al. (2021) **[205]**,** **Sweden (Swedish BioFINDER-2)**	Cross-sectional and subset with longitudinal, non-fasting, EDTA plasma, − 80 °C	Aβ PET and MMSE score	CU/Aβ negative: *n* = 217 CU/Aβ positive: *n* = 71 CI/Aβ negative: *n* = 63 CU/Aβ positive: *n* = 78 Non AD: *n* = 75 Covariates: age and sex	SIMOA GFAP Discovery kits (Quanterix, Billerica, MA, USA)	NA	Plasma GFAP holds great potential as an early and specific marker of amyloid-β deposition, even during the earliest stages of AD
**Elahi et al. (2020) **[27]**,** **USA (UCSF Memory and Ageing Center)**	Cross-sectional, EDTA plasma, − 80 °C	NIA-AAA, Aβ PET	Early-onset AD, 33 Late-onset AD, 30 HC, 33 Covariate: age	SIMOA GFAP Discovery kits (Quanterix, Billerica, MA, USA)	Duplicated samples, < 20% CV considered	Elevation in plasma GFAP in early-onset AD was observed compared to control and, to a lesser extent, in late-onset AD
**Chatterjee et al. (2023) **[33]**,** **Australia (KARVIAH cohort)**	Cross-sectional with 12-month follow-up, fasting plasma, EDTA, − 80 °C	Aβ PET, MRI, and MMSE score	CU/Aβ negative: *n* = 67 CU/Aβ positive: *n* = 33 Covariates: age, sex, and APOE ε4 status	Neurology 4-Plex A kit (Quanterix®, Billerica, MA, USA)	Calibrators and samples were duplicated Two levels QC used; LOD, 0.467 pg/mL; CV, 2.72%	Plasma GFAP combined with the AD risk factors had the highest accuracy in differentiating between cognitively unimpaired older adults (CU) with amyloidosis (Aβ positive) and without amyloidosis (Aβ negative), indicating its potential as a diagnostic marker for preclinical AD
**Gonzales et al. (2022) **[31]**,** **USA (Mexican American cohort and Texas Alzheimer’s Research and Care Consortium study)**	Longitudinal community based (4 years), non-fasting serum, − 80 °C	NINCDS-ADRDA	CU: *n* = 479 MCI: *n* = 207 AD: *n* = 59 Covariates: age, sex, ethnicity, APOE ε4 status, education, blood pressure, hypertension, diabetic mellitus, BMI	Neurology 4-Plex kit (Quanterix, Billerica, MA, USA)	Analytical ranges (0.8–3 420 pg/mL) and inter-assay coefficients of variance (13.9%) reported	Higher baseline levels of GFAP were linked to an increased risk of incident dementia due to possible/probable AD
**Beyer et al. (2022) **[81]**,** **Germany (ESTHER cohort)**	Prospective cohort (17 years), lithium-heparin plasma, − 80 °C	NINCDS–ADRDA	AD: *n* = 68 Control: *n* = 208 Covariates: age, sex, APOE ε4 status	Neurology 4-Plex E Advantage kits (Quanterix, Billerica, MA, USA)	NA	GFAP levels were significantly higher in participants who were diagnosed with AD within 17 years
**Cicognola et al. (2021) **[209]**,** **Sweden (Memory Clinic)**	Longitudinal (4.7 years), non-fasting, EDTA plasma, − 80 °C	NINCDS-ADRDA Aβ positive: CSF Aβ_42_/Aβ_40_ < 0.07 pg/mL Aβ negative: CSF Aβ_42_/Aβ_40_ > 0.07 pg/mL	MCI: *n* = 160 MCI no dementia: *n* = 79 MCI-AD: *n* = 47 MCI other dementia: *n* = 34 Vascular dementia: *n* = 25 DLB: *n* = 4 PSP: *n* = 3 Covariates: age and sex	SIMOA GFAP Discovery kits (Quanterix, Billerica, MA, USA)	NA	Baseline high GFAP concentration was a strong indicator of AD pathology
**Gonzales et al. (2022) **[220]**,** **USA (FHS cohort, CHS, CARDIA cohorts) and Iceland (AGES)**	Community-based longitudinal retrospective cohorts, fasting plasma and serum, − 80 °C	DSM IV, NINCDS-ADRDA	All cases of dementia: *n* = 554 AD: *n* = 390 Covariates: age, sex, education, race, diabetes, systolic blood pressure, antihypertensive medication use, body mass index, APOE ε4 status	Neurology 4-Plex A kit (Quanterix, Billerica, MA, USA)	Analytical range: 4.64–3784 pg/mL; inter-assay CV, 9.7%	Elevations in baseline GFAP levels were strongly associated with an increased risk of incident dementia over the up to 15‐year follow‐up period
**Bucci et al. (2023) **[221]**,** **Sweden (the Clinic for Cognitive Disorders at Karolinska University Hospital in Stockholm)**	Retrospective clinical cohort, plasma collected into sodium-heparin tubes, − 80 °C	NIA-AA, Aβ PET	MCI Aβ − : *n* = 29, MCI Aβ positive: *n* = 19 AD: *n* = 51 Non-AD dementia: *n* = 23 No dementia: *n* = 4 Covariates: age, sex,	Neurology 4-Plex E (Quanterix, Billerica, MA, USA)	NA	Plasma GFAP levels were associated with Aβ PET centiloid levels in the MCI group
**Varma et al. (2025) **[213]**,** **USA (The Baltimore Longitudinal Study of Aging by the National Institute on Aging)**	Prospective, observational cohort	Confirmation of AD pathology at autopsy, determined by an expert neuropathologist	AD converters (*n* = 158), cognitively unimpaired (*n* = 160)	Neurology 4-Plex E (Quanterix, Billerica, MA, USA)	Inter-assay CV, 8.1%	Elevated plasma GFAP levels have been observed 10 years prior to the clinical onset of cognitive decline due to AD
**Abbas et al. (2025) **[218]**,** **Canada (TRIAD cohort), USA and Canada (ADNI), and Korea (BICWALZS)**	Longitudinal observed (≤ 2.5 years follow-up duration)	Global clinical dementia rating score and Aβ PET	TRIAD cohort: *n* = 109 (CU/Aβ negative, *n* = 53; CU/Aβ positive, *n* = 18; CI/Aβ negative, *n* = 13; CI/Aβ positive, *n* = 25) ADNI: *n* = 223 (CU/Aβ negative, *n* = 73; CU/Aβ positive, *n* = 32; CI/Aβ negative, *n* = 68; CI/Aβ positive, *n* = 50) BICWALZS: *n* = 155 (CU/Aβ negative, *n* = 0; CU/Aβ positive, *n* = 0; CI/Aβ negative, *n* = 73; CI/Aβ positive, *n* = 82)	Neurology 4-Plex E (Quanterix, Billerica, MA, USA)	ADNI: intra-assay CV, 4.9%	Plasma GFAP levels were increased parallel to cognitive decline
**Plasma GFAP associated with cerebral Aβ load**						
**Asken et al. (2020) **[207]**,** **USA (UCSF)**	Cross-sectional, plasma, − 80 °C	Multidisciplinary consensus conference (neurologic exam, cognitive testing, and clinical dementia rating)	Cohort 1: *n* = 50 (HC, *n* = 39; MCI, *n* = 11; dementia, *n* = 0) Cohort 2: *n* = 37 (HC, 14; MCI, 11; dementia, 12) Covariates: age and sex	Neurology 4-Plex A kit (Quanterix, Billerica, MA, USA)	NA	Plasma GFAP increased linearly with Aβ-PET centiloids in older adults with mild or no functional changes, primarily driven by functionally intact participants Aβ-GFAP association became curvilinear—plasma GFAP concentrations decreased as Aβ-PET exceeded 100 centiloids
**Cicognola et al. (2021) **[209]**,** **Sweden (Memory Clinic)**	Longitudinal (4.7 years), non-fasting, EDTA plasma, − 80 °C	NINCDS-ADRDA Aβ positive: CSF Aβ < 0.07 Ab negative: CSF Ab > 0.07	MCI: *n* = 160 MCI no dementia: *n* = 79 MCI-AD: *n* = 47 MCI other dementia: *n* = 34 Vascular dementia: *n* = 25 DLB: *n* = 4 PSP: *n* = 3 Covariates: age and sex	SIMOA GFAP Discovery kits (Quanterix, Billerica, MA, USA)	NA	Differences at baseline seemed to be associated with (and possibly driven by) the Aβ status; higher concentrations at baseline were observed in every Aβ-positive subgroup compared to Aβ-negative ones
**Shir et al. (2022) **[29]**,** **USA (Mayo Clinic study of ageing)**	Cross-sectional, overnight fasting plasma, − 80 °C	DSM IV	Aβ positive: *n* = 99 Aβ negative: *n* = 101 Covariates: age, sex, and years of education, APOE ε4 status	Neurology 4-Plex E Advantage kits (Quanterix, Billerica, MA, USA)	Two levels QC, inter-assay CV (235 pg/mL. 7.2%; 5055 pg/mL, 8.3%)	Plasma GFAP levels in persons without dementia are associated with Aβ PET burden
**Chatterjee et al. (2022) **[32]**,** **Australia (AIBL cohort)**	Cross-sectional and longitudinal (3 years), EDTA plasma, − 80 °C	NINCDS-ADRDA criteria	CU/Aβ negative: *n* = 81 MCI/Aβ negative: *n* = 26 CU/Aβ positive: *n* = 39 MCI/Aβ positive: *n* = 33; AD, 46 Covariates: age, sex, APOE ε4 carrier status	Neurology 4-Plex A kit (Quanterix, Billerica, Massachusetts, USA)	Samples in singlicates, calibrators duplicate, positive QC and CV measured (3.26%)	Longitudinally, GFAP is associated with increased PET Aβ load prospectively
**Bucci et al. (2023) **[221]**,** **the Clinic for Cognitive Disorders at Karolinska University Hospital in Stockholm, Sweden**	Retrospective clinical cohort, plasma collected into sodium-heparin tubes, − 80 °C	NIA-AAA, Aβ PET	MCI Aβ − : *n* = 29, MCI Aβ + : *n* = 19 AD: *n* = 51, non-AD dementia: *n* = 23, no dementia: *n* = 4 Covariates: age and sex	Neurology 4-Plex E (Quanterix, Billerica, MA, USA)	NA	Higher plasma GFAP levels were observed in amyloid-positive MCI individuals compared to amyloid-negative MCI individuals
**Plasma GFAP is a useful marker to differentiate the stages of AD**						
**Parvizi et al. (2022) **[210]**,** **Austria (Heterogenous Memory Clinic, Medical University of Vienna)**	Retrospective study, EDTA plasma, − 80 °C	NIA-AA and subset with biomarker approach (CSF or Aβ PET or both)	HC: *n* = 44 MCI: *n* = 63 AD: *n* = 60	Neurology 2-Plex B kit (Quanterix, Billerica, MA, USA)	Inter- (< 12%) and intra-assay CV (< 12%) reported	The plasma GFAP gradually increased along with the disease severity, with the most prominent difference being MCI and HC
**Chatterjee et al. (2022) **[32]**,** **Australia** **(AIBL cohort)**	Cross-sectional and longitudinal (3 years), EDTA plasma, − 80 °C	NINCDS-ADRDA	CU/Aβ negative: *n* = 81, MCI/Aβ negative: *n* = 26 CU/Aβ positive: *n* = 39, MCI/Aβ positive: *n* = 33; AD, 46 Covariates: age, sex, and APOE ε4 carrier status	Neurology 4-Plex A kit (Quanterix, Billerica, Massachusetts, USA)	Samples in singlicates , calibrator duplicates, positive QC and CV measured (3.26%)	Longitudinally, GFAP was altered in MCI versus CU and AD versus CU
**Plasma GFAP as a prognostic biomarker**						
**Gonzales et al. (2022) **[220]**,** **USA (FHS cohort, CHS, CARDIA cohorts) and Iceland (AGES)**	Community-based longitudinal retrospective cohorts, fasting plasma and serum, − 80 °C	DSM IV, NINCDS-ADRDA	All cases of dementia: *n* = 554, AD: *n* = 390 Covariates: age, sex, education, race, diabetes, systolic blood pressure, antihypertensive medication use, body mass index, and APOE ε4 status	Neurology 4-Plex A kit (Quanterix, Billerica, MA, USA)	Analytical range: 4.64–3784 pg/mL; inter-assay CV, 9.7%	Higher circulating GFAP was associated with poorer general cognition but not total brain or hippocampal volume
**Gonzales et al. (2022) **[31]**,** **USA (Mexican American cohort, Texas Alzheimer’s Research and Care Consortium study)**	Longitudinal community-based (4 years), non-fasting serum, − 80 °C	NINCDS-ADRDA	CU, 479 MCI, 207 AD, 59 Covariates: age, sex, ethnicity, APOE ε4 status, education, blood pressure, hypertension, diabetic mellitus, and BMI	Neurology 4-Plex kit (Quanterix, Billerica, MA, USA)	Analytical ranges (0.8–3420) and inter-assay coefficients of variance (13.9%) reported	Higher baseline GFAP levels were associated with accelerated cognitive decline
**Verberk et al. (2021) **[28]**,** **Netherlands (Amsterdam Dementia cohort)**	Prospective cohort (2.6 years), non-fasting serum, − 80 °C	Brain MRI, APOE, and CSF Aβ_42_ by ELISA (< 813 pg/mL) Covariates: age and sex were adjusted for the clinical progression of dementia; age, sex, and education were adjusted for neuropsychological performance	No dementia: *n* = 273 AD: *n* = 27	SIMOA GFAP Discovery Kit (Quanterix, Billerica, MA, USA)	Sample duplicated within 3% CV; 3 quality control samples were used, and the assay was in-house validated	Higher GFAP levels were related to steeper rates of cognitive decline in the domains of memory, attention, and executive functioning
**Bettcher et al. (2021) **[173]**,** **USA (University of Colorado Alzheimer’s and Cognition Center database)**	Cross-sectional, plasma, − 80 °C	NIA-AAA	Asymptomatic: *n* = 69 Symptomatic: *n* = MCI AD = 45 Covariates: age, gender, APOE ɛ4 status, and education	SIMOA GFAP Discovery kit (Quanterix, Billerica, MA, USA)	Intra-individual CV (< 20%) was reported	Higher blood levels of a GFAP were associated with worse memory performance and poorer microstructural integrity of medial temporal white matter tracts
**Chatterjee et al. (2022) **[32]**,** **Australia (AIBL cohort)**	Cross-sectional and longitudinal (03 years), EDTA plasma, − 80 °C	NINCDS-ADRDA criteria	Aβ negative: CU, 81; MCI, 26 Aβ positive: CU, 39; MCI, 33; AD, 46 Covariates: age, sex, and APOE ε4 carrier status	Neurology 4-Plex A kit (Quanterix, Billerica, Massachusetts, USA)	Samples in singlicates, calibrator duplicates, positive QC and CV measured (3.26%)	Longitudinally, GFAP was associated with prospective cognitive decline
**Oeckl et al. (2019) **[211]**,** **Germany**	Cross-sectional, Serum, − 80 °C, 5 freeze–thaw cycles	NIA-AAA and CSF tau and Aβ_42_ levels	AD: *n* = 28 FTD: *n* = 35 PD: *n* = 11 DLB: *n* = 19 HC: *n* = 34 Covariates: age	Human GFAP Discovery kit (Quanterix, Lexington, MA, USA)	Inter (< 2%) and Intra (< 11%) assay variability reported	Serum GFAP correlated with MMSE score
**Shir et al. (2022) **[29]**,** **USA (Mayo Clinic study of aging)**	Cross-sectional population‐based study, overnight fast plasma, − 80 °C	DSM IV	Aβ positive: *n* = 99 Aβ negative: *n* = 101 Covariates: age, sex, years of education, and APOE ɛ4 status	Neurology 4-Plex E Advantage Kits (Quanterix, Billerica, MA, USA)	Two-level QC, inter-assay CV (at 235 pg/mL, 7.2%; at 5055 pg/mL, 8.3%)	Plasma GFAP was associated with decreased cortical thickness, increased white matter hyperintensities, and cerebral microbleeds among amyloid-positive individuals
**Plasma GFAP in AD compared to other neurodegenerative dementia types**						
**Baiardi et al. (2022) **[222]**,** **Italy (neuropathology laboratory (NP-Lab) at the Institute of Neurological Science of Bologna)**	Retrospective analysis, EDTA plasma, − 80 °C	NIA-AA	FTD: *n* = 59 PSP: *n* = 31 CBS: *n* = 29 DLB: *n* = 49 AD: *n* = 97 Non-AD: *n* = 51 HC: *n* = 51	SIMOA GFAP Discovery kit (Quanterix, Billerica, MA, USA)	Inter- (9%) and intra-assay (12%) CV reported	Plasma GFAP distinguished AD more efficiently against FTD (AUC 0.818) and PSP (AUC 0.765) than against CBS (AUC 0.616) and DLB (AUC 0.578)
**Oeckl et al. (2019) **[211]**,** **Germany**	Cross-sectional, serum, − 80 °C, 5 freeze–thaw cycles	NIA-AAA and CSF tau and Aβ_42_ levels	AD: *n* = 28 FTD: *n* = 35 PD: *n* = 11 DLB: *n* = 19 HC: *n* = 34 Covariate: age	Human GFAP Discovery kit (Quanterix, Lexington, MA, USA)	Inter- (< 2%) and Intra-assay (< 11%) variability reported	Serum GFAP was increased in AD and DLB/PDD, and cerebrospinal fluid GFAP was increased in all neurodegenerative diseases
**Bucci et al. (2023) **[221]**,** **the Clinic for Cognitive Disorders at Karolinska University Hospital in Stockholm, Sweden**	Retrospective clinical cohort, plasma collected into sodium-heparin tubes, − 80 °C	NIA-AAA, Aβ PET	MCI Aβ − : *n* = 29, MCI Aβ + : *n* = 19 AD: *n* = 51, non-AD dementia: *n* = 23, no dementia: *n* = 4 Covariates: age, sex	Neurology 4-Plex E (Quanterix, Billerica, MA, USA)	NA	Plasma GFAP levels were elevated in AD compared to non-AD dementia individuals

*AD* Alzheimer’s disease, AGES age, gene/environment susceptibility–Reykjavik study, *ADNI* Alzheimer’s disease neuroimaging initiative, *AIBL* Australian imaging, biomarker and lifestyle, *ALFA* + Alzheimer’s and families, *APOE* apolipoprotein E, *Aβ* amyloid beta, *BICWALZS* biobank innovations for chronic cerebrovascular disease with Alzheimer’s disease study,* BMI* body mass index, *CARDIA* coronary artery risk development in young adults, *CBS* corticobasal syndrome, *CHS* cardiovascular health study, *CI* cognitively impaired, *CSF* cerebrospinal fluid, *CU* cognitively unimpaired, *CV* coefficient of variation, *DLB* dementia with Lewy bodies, *DSM-IV* Diagnostic and Statistical Manual of Mental Disorders, 4th Edition, *EDTA* ethylenediaminetetraacetic acid, *ELISA* enzyme-linked immunosorbent assay, *ESTHER* epidemiological investigations of the chances of preventing, recognising early and optimally treating chronic diseases in an elderly population, *FHS* Framingham heart study, *FTD* frontotemporal degeneration, *GFAP* glial fibrillary acidic protein, *HC* healthy control, IWG-2 International Working Group, *KARVIAH* Kerr Anglican retirement village initiative in ageing health cohort, *MCI* mild cognitive impairment, *MMSE* mini mental state examination, *MRI* magnetic resonance imaging, NA not available, *NIA-AA* National Institute on Aging and the Alzheimer’s Association, *NINCDS-ADRDA* National Institute of Neurological and Communicative Disorders and Stroke–Alzheimer’s Disease and Related Disorders Association, PDD Parkinson’s disease dementia, *PET* positron emission tomography,* PSP* progressive supranuclear palsy, *QC* quality control, SIMOA single molecule array, *SUVR* standardised uptake value ratio, *TRIAD* translational biomarkers in ageing and dementia, *UCSF* University of California, San Francisco

### Comparison Between Plasma and CSF GFAP Levels in AD

The plasma GFAP levels could more accurately differentiate Aβ-positive from Aβ-negative individuals compared to CSF GFAP [139, 205, 208]. Additionally, plasma GFAP consistently shows larger effect sizes than CSF GFAP, reinforcing its superior utility as a peripheral biomarker for Aβ pathology. In contrast, CSF GFAP levels only increase significantly in patients with Aβ and tau pathology, suggesting that plasma GFAP may reflect earlier pathological changes than CSF GFAP [208, 211]. However, Simrén et al. [223] observed that the pre-analytical sample preparation reduced the CSF GFAP levels.

One major challenge in CSF GFAP measurement is sample stability. Plasma GFAP remains relatively stable after multiple freeze–thaw cycles [224]. In contrast, CSF GFAP levels decline with repeated freezing and thawing, raising concerns about pre-analytical variability in CSF-based assays [223]. The differences in GFAP stability may be due to variations in its clearance mechanisms between blood and CSF. Astrocytic GFAP enters circulation through the BBB, blood-CSF barrier, and the lymphatic system [130, 225]. BBB disruption in AD may enhance GFAP leakage into the bloodstream, making plasma GFAP a more accessible and reflective indicator of Aβ pathology [226, 227].

### Plasma GFAP in Differentiating AD from Other Neurodegenerative Dementias

Few studies have directly compared plasma GFAP levels with other neurodegenerative dementias. For example, plasma GFAP has demonstrated high effectiveness in distinguishing AD from frontotemporal dementia and progressive supranuclear palsy, suggesting its potential as a CSF-independent biomarker [211, 222]. However, its ability to differentiate AD from corticobasal syndrome and dementia with Lewy bodies is less effective, limiting its use in distinguishing certain atypical dementia syndromes [211].

Similar to CSF findings, plasma GFAP is elevated across multiple neurodegenerative diseases, including frontotemporal dementia, Parkinson’s disease dementia, dementia with Lewy bodies, and AD. However, plasma GFAP levels are significantly higher in AD and dementia with Lewy bodies compared to other dementias [211, 222], suggesting that GFAP may be particularly useful in differentiating AD from frontotemporal dementia and Parkinson’s disease dementia. Nevertheless, further studies are needed to establish disease-specific GFAP cut-off values for improved clinical utility.

### Advancements in Blood-Based GFAP Measurement Technologies

Most studies measuring blood GFAP levels in AD have utilised Single Molecule Array (SIMOA) technology (Table 3.), the most widely adopted ultra-sensitive detection method. However, several novel technologies have emerged for measuring GFAP and other blood-based biomarkers, including Immunoprecipitation Liquid Chromatography-Mass Spectrometry [228, 229], SomaScan [230, 231], Olink [230, 232–234], Nucleic Acid Linked Immuno-Sandwich Assay [235], Lumipulse [236–238], Mesoscale Discovery [239, 240], and NeuroToolKit (Roche Diagnostics’ cobas Elecsys assays) [241].

Studies comparing different GFAP measurement techniques have found a strong correlation between Nucleic Acid Linked Immuno-Sandwich Assay and SIMOA plasma GFAP levels, with SIMOA demonstrating higher predictive power for AD diagnosis [242]. However, comparative performance studies on GFAP across novel technologies remain limited, as most research has focused on Aβ and tau biomarkers [234, 236].

Future studies should evaluate GFAP measurement across multiple platforms to determine the most accurate and reliable detection method for clinical use. Further validation is required to assess GFAP’s consistency across different blood-based diagnostic assays to improve its clinical applicability in AD and other neurodegenerative diseases.

## The Influence of Biological Variation and Lifestyle Factors on Blood GFAP in AD

Blood GFAP levels in AD are influenced by biological factors such as age and sex, as well as lifestyle-related variables. While some lifestyle modifications have been linked to reduced AD risk, their direct impact on plasma GFAP levels remains unclear. Understanding how these factors interact with astrocyte reactivity and GFAP expression is crucial for improving biomarker interpretation and disease risk assessment.

### Biological Factors Affecting Plasma GFAP in AD

Age-related changes in GFAP expression and astrogliosis have been reported in healthy individuals, suggesting that astrocytic alterations occur as part of normal ageing, independent of AD pathology. Studies have shown a progressive increase in GFAP mRNA expression and protein levels with age. This may contribute to higher baseline plasma GFAP levels in older adults, even without neurodegenerative disease [243]. This highlights the need for age-adjusted reference values when interpreting plasma GFAP as a biomarker for AD.

Sex differences have also been observed, with female participants consistently showing higher plasma GFAP levels than males. This difference has been linked to increased astrocyte reactivity in women, as measured by GFAP expression. Studies reported a significant correlation between plasma GFAP levels and hippocampal volume in women but not in men, suggesting that astrocytic responses may vary by sex in the context of AD [244, 245]. These findings emphasise the importance of considering sex-specific variations in GFAP levels when assessing its role as a biomarker. Beyond age and sex, ethnic background may also contribute to biological variations in GFAP levels. Only one study has investigated plasma GFAP variation among subgroups within the Hispanic population, while most research has exclusively focused on Caucasian individuals [31]. The lack of diverse population data limits our understanding of ethnicity-based differences in astrocytic responses and GFAP biomarker interpretation. Investigating these gaps further may contribute to developing more inclusive and representative biomarker reference values.

### Lifestyle Factors and Their Potential Influence on Plasma GFAP

Since sporadic AD cases account for the majority of diagnoses, lifestyle modifications are often considered preventative strategies. Various interventions—including nutritional changes, physical activity, cognitive training, and sleep regulation—have been proposed to reduce AD risk [39, 246–250]. However, their direct impact on plasma GFAP levels remains largely unexplored.

#### Diet and Nutrition

Specific dietary patterns, particularly the Mediterranean diet (rich in omega-3 fatty acids and polyphenol-rich foods, olive oil, nuts, vegetables and fruits) and MIND diet (a combination of the Mediterranean diet and Dietary Approaches to Stop Hypertension intervention diets for neuroprotection) have been associated with reduced risk of cognitive decline and cerebral Aβ accumulation over time [251, 252]. However, studies investigating their effect on plasma GFAP levels have been inconclusive. A 1-year nutritional intervention study that included various vitamins, antioxidants, and essential fatty acids found no significant impact on plasma GFAP levels in participants receiving supplementation compared to a placebo group [253]. Even among APOE ε4 carriers with higher baseline GFAP and p-Tau 181 levels, no significant differences were observed after the intervention. Interestingly, plasma GFAP raises questions about whether astrocytic reactivity is influenced by ageing rather than dietary supplementation [253]. Additionally, another study found no association between plasma GFAP and adherence to the Mediterranean diet among individuals on the AD continuum, suggesting that dietary components alone may not directly regulate GFAP expression [254].

#### Physical Activity and Plasma GFAP

The effects of physical activity on plasma GFAP in MCI and AD patients remain poorly understood, as few studies have specifically examined this association with memory [255, 256]. The limited research has focused on cognitively unimpaired older adults, where no significant changes in plasma GFAP levels were observed following exercise interventions [256]. Another study found no association between physical activity and memory performance with GFAP [255]. Similarly, a 6-month exercise intervention study in cognitively unimpaired older adults found no significant changes in plasma GFAP levels following moderate-to-high-intensity cycling training [256]. However, whether more extended follow-up periods would reveal exercise-induced effects on GFAP levels remains unclear.

Given the well-established benefits of physical activity in reducing AD risk and promoting brain health, future research should explore whether exercise modulates astrocytic reactivity and GFAP levels over extended periods. This is particularly important as astrocyte activation is believed to be an early pathological event in AD.

#### Sleep Quality and Cognitive Training

The relationship between sleep quality and plasma GFAP is another growing interest. Sleep disruptions have been identified as a risk factor for AD [250, 257], with some studies linking poor sleep quality to increased astrocyte reactivity [258, 259]. Only one clinical study showed elevated GFAP levels as an indicator of disrupted sleep and sleep‐related disorders in individuals at risk for AD—in particular, for APOE ε4 carriers with sleep disorder who had the highest plasma GFAP compared to non-carriers with or without sleep disorder [260]. These findings suggest that disrupted sleep may accelerate astrocyte activation and contribute to early AD pathology.

However, research on cognitive training and its effects on plasma GFAP remains scarce. While cognitive training has been associated with improved cognitive outcomes and delayed AD onset, no studies have established whether it influences plasma GFAP levels or astrocyte function. Future studies should investigate whether cognitive engagement and brain stimulation alter GFAP expression in individuals at risk for AD.

## Research Gaps, Limitations, and Future Directions

While plasma biomarkers have shown promise in detecting Aβ and tau pathology in AD, several gaps in the literature and methodological limitations must be addressed before blood GFAP can be fully integrated as a clinical biomarker. Existing research highlights the potential of plasma GFAP as a predictive marker for identifying individuals at risk for AD in the preclinical stage. However, key issues related to study design, analytical variability, and the biological significance of GFAP in AD pathology remain unresolved.

### Limitations in Study Design and Population Diversity

Most studies on plasma GFAP in AD have been single-centre cross-sectional studies (Table 3.) with small sample sizes and heterogeneous diagnostic procedures. Many lacked comprehensive neuroimaging assessments (MRI, CSF, and PET imaging outcomes), limiting their ability to correlate GFAP changes with structural and pathological markers of AD progression. Future studies should prioritise extensive, longitudinal, multi-centre studies with sociodemographically diverse populations to validate existing findings.

Lack of population diversity remains a significant limitation, as nearly all studies have focused on Caucasian individuals, with only one study investigating plasma GFAP levels in Hispanic populations. Sex differences have also been underexplored despite evidence suggesting that women with high Aβ burden exhibit significantly higher GFAP levels than men with similar pathology. Future studies must incorporate sex- and ethnicity-specific analyses to ensure the reliability of GFAP as a biomarker across diverse populations.

### Analytical Variability and Standardisation Challenges

One of the major barriers to clinical translation is the variability in GFAP measurement due to differences in sample collection, handling, and storage. Studies have used plasma and serum samples collected in ethylenediaminetetraacetic acid (EDTA) or lithium-heparin tubes and stored at − 80 °C before analysis. While plasma GFAP has demonstrated greater stability across multiple freeze–thaw cycles compared to CSF GFAP, variations in pre-analytical sample preparation can still introduce inconsistencies in biomarker levels [223, 224, 261]. Similar to plasma NFL, plasma GFAP levels had relatively high between-subject variation, which impacts depend on clinical context [262]. Other biological factors, including circadian rhythm and post-prandial food intake, have been observed as significant factors in plasma GFAP levels [263]. While some studies have controlled for these variables by collecting overnight fasting plasma samples, many did not, leading to potential discrepancies in findings. Additionally, most studies did not comprehensively report a history of head trauma, other clinical confounders, and peripheral neuropathies, which may influence plasma GFAP levels independent of ageing and AD when selecting participants in the studies [207]. Establishing standardised pre-analytical protocols and reporting criteria is crucial for improving the reliability and reproducibility of GFAP measurements in clinical settings.

### Lack of Standardised Immunoassays and GFAP Isoform and PTMs Characterisation

Most AD studies measuring plasma GFAP have relied on Quanterix SIMOA-based assays, including GFAP Discovery (Quanterix, USA), Neurology 4-Plex A, Neurology 4‐Plex E, and Neurology 2-Plex B kits (Quanterix, USA) [30, 32, 264]. While these platforms offer high sensitivity, a major limitation is the lack of standardisation across different immunoassays, making cross-study comparisons challenging. The specific GFAP epitopes these antibodies target are often unknown, limiting our understanding of whether they detect full-length GFAP, GFAP breakdown products, GFAP isoforms, or PTM GFAP. However, the epitopes of GFAP antibodies in commercially available kits are usually unknown, limiting the knowledge of the isoforms these antibodies target, apart from the entire length of GFAP [134]. The absence of well-characterised GFAP isoforms in plasma and CSF remains a significant knowledge gap. Unlike Aβ_42_/Aβ_40_ and phosphorylated tau biomarkers, which have well-defined cut-off ratios for AD diagnosis, it is unclear whether different GFAP isoforms or PTMs provide better diagnostic and prognostic value than total GFAP levels. Future research should focus on identifying AD-specific GFAP isoforms, characterising their post-translational modifications, and determining whether they correlate with disease severity.

### The Need for Improved Assay Sensitivity and Specificity

While plasma GFAP has demonstrated a strong association with Aβ pathology, it has shown no clear relationship with fibrillar tau accumulation. This suggests that plasma GFAP alone may not be sufficient for staging AD pathology and should be validated alongside other biomarkers to elucidate its precise role in astrocytic dysfunction and neurodegeneration. Given the growing recognition of GFAP as a biomarker for multiple neurodegenerative diseases, it is essential to refine analytical approaches to differentiate AD-specific astrocyte responses from those occurring in other conditions. The development of AD-specific GFAP immunoassays should consider the following:

- Targeting GFAP isoforms that are preferentially upregulated in AD.
- Accounting for GFAP PTMs that may serve as better diagnostic markers, similar to how phosphorylated tau has improved AD diagnostics.
- Utilising antibody pairs that distinguish full-length GFAP from GFAP-BDP, as GFAP-BDP levels may provide additional insights into astrocyte reactivity and disease progression.

A promising approach to enhance the sensitivity and specificity of GFAP immunoassays, such as SIMOA, for AD diagnosis is to consider all GFAP isoforms, PTMs, and GFAP-BDP during antibody design and selection. Optimising antibody pairing by targeting distinct GFAP epitopes, including those associated with AD-specific GFAP isoforms and those less influenced by PTMs, may significantly improve the accuracy of AD-specific immunoassays.

It is possible that the ratio of specific GFAP isoforms could serve as a more effective biomarker for distinguishing AD from cognitively normal individuals, similar to the diagnostic utility of the Aβ_42_/Aβ_40_ ratio [265]. Likewise, certain GFAP PTMs, such as phosphorylated GFAP, may provide better differentiation between disease stages, like phosphorylated tau in AD [266].

To further advance the development of AD-specific SIMOA assays, immunoprecipitation-mass spectrometry offers a valuable tool for identifying GFAP species beyond full-length GFAP, including isoforms, PTMs, and GFAP-BDP, by comparing the plasma GFAP between AD and other neurodegenerative diseases. This approach can identify what forms of GFAP are dominant in AD, enabling the development of specific GFAP antibodies to target it.

### GFAP Research and Clinical Applications

Further research is needed to explore the relationship between GFAP and other AD biomarkers, particularly phosphorylated tau and NFL, to determine whether combined biomarker panels provide better diagnostic accuracy than single-marker assessments. Additionally, longitudinal studies should examine how plasma GFAP levels change with disease progression, cognitive decline, structural brain changes, and underlying astrocyte heterogeneity.

Another critical gap in the literature is the lack of data on lifestyle-based interventions and their influence on plasma GFAP levels. While modifiable risk factors such as diet, physical activity, sleep quality, and cognitive engagement have been shown to delay cognitive decline and reduce AD risk, their impact on GFAP expression remains largely unexplored. Future clinical trials should investigate whether specific lifestyle modifications can modulate astrocyte reactivity and GFAP levels, offering potential non-pharmacological AD prevention and management strategies.

## Conclusion

Plasma GFAP has emerged as a promising biomarker for AD, particularly in the early detection of Aβ pathology and disease progression. Elevated plasma GFAP levels have been consistently associated with preclinical and symptomatic AD, demonstrating strong correlations with Aβ deposition and cognitive decline. However, despite its potential, several critical challenges remain before it can be fully integrated into clinical diagnostics. Methodological inconsistencies, analytical variability, and a lack of standardisation across studies have hindered the reproducibility and clinical application of plasma GFAP measurements. Factors such as pre-analytical sample handling, biological variability, and differences in immunoassay platforms contribute to discrepancies in findings. Sex-specific differences, population diversity, and comorbid conditions further complicate the interpretation of GFAP levels, emphasising the need for large, multi-centre longitudinal studies with diverse cohorts to validate its role as a diagnostic and prognostic biomarker. Moreover, the specificity of GFAP for AD needs further evaluation, as elevated plasma levels have been observed in various neurodegenerative diseases. While plasma GFAP shows a stronger association with cerebral Aβ pathology than with tau pathology, further studies are required to understand its precise role in AD pathophysiology. Investigating GFAP isoforms, PTMs, and fragmentations may provide greater diagnostic accuracy and disease staging capabilities, similar to the established Aβ_42_/Aβ_40_ and phosphorylated tau biomarkers. GFAP immunoassays that target AD-specific GFAP isoforms and utilise different platforms, such as SIMOA, Nucleic Acid Linked Immuno-Sandwich Assay, SomaScan, Olink, and immunoprecipitation-mass spectrometry, can significantly increase the sensitivity and specificity of GFAP-based diagnostics in the future. Additionally, exploring the impact of lifestyle interventions, including diet, physical activity, cognitive engagement, and sleep quality, on plasma GFAP levels may offer novel insights into astrocytic function and disease modulation.

## Data Availability

No datasets were generated or analysed during the current study.
